# Different co-culture approaches to increase metabolites in foods

**DOI:** 10.1007/s10068-026-02160-6

**Published:** 2026-04-17

**Authors:** Günes Selay Uysal, Hatice Kalkan Yıldırım

**Affiliations:** Department of Food Engineering, Faculty of Engineering, Ege University, Bornova, 35100 Izmir, Türkiye

**Keywords:** Co-culture, Microbial interaction, Microbial communication, Modular co-culture, Food metabolite

## Abstract

Microbial co-cultures have emerged as effective methods for enhancing metabolite production, which surpass the limitations of traditional monoculture approaches by offering enhanced efficiency, lower costs, better stress resistance and more sophisticated processes execution. Co-cultivation strategies, such as microbial interactions, modular co-culture, and microbial communication, are gaining attention for their potential to enhance food-related metabolites. Nevertheless, studies investigating these methods directly within food matrices are significantly scarce. Therefore, this review highlighted various co-cultivation approaches for metabolite enhancement in foods, along with the challenges and future perspectives. The importance of using co-culture methodologies for increasing food metabolite production was demonstrated in this study. It was also indicated that co-culture systems still encounter problems like system complexity, stability and scale-up. Future research should prioritize combining advanced technologies with co-culture systems to overcome challenges, and applying co-culture approaches, especially engaging multi-strains, directly into the food matrices to uncover the whole potential of these strategies.

**Graphical Abstract:**

**Figure:**
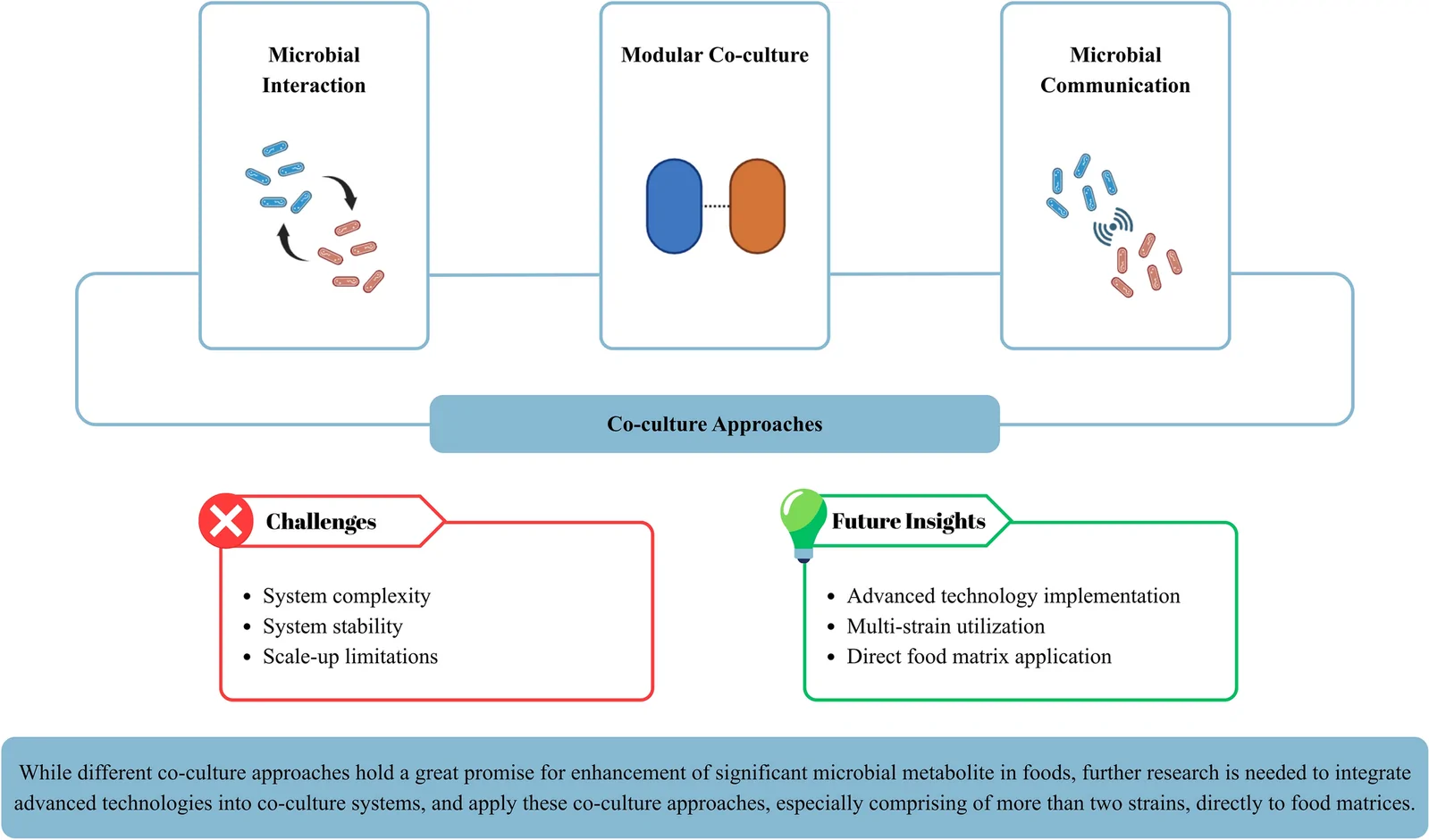

## Introduction

Microorganisms have been considered as important sources of organic compounds which are called microbial metabolites (Tan et al., 2019). Microorganisms and the metabolites they produce contribute significantly to fields such as agriculture (Nadarajah and Abdul Rahman, 2023), food (Smid and Lacroix, 2013), drug (Selegato and Castro-Gamboa, 2023) and environment (Ayilara and Babalola, 2023) by counteracting difficulties in agroecology, food safety, human health and environmental remediation (Dinglasan et al., 2025; Sokołowska, 2024). Microbial metabolites, which are often characterized by their small molecular size, are the intermediate and final compounds produced during metabolic processes of microorganisms (Ofoedum et al., 2024). These metabolites are produced by a wide range of microorganisms including bacteria, fungi, cyanobacteria and yeasts. Besides being essential for human life, they are also important for the survival of microorganisms, their adaptation to environmental conditions, and their interactions with other microorganisms (Altaf-Ul-Amin et al., 2019; Guo et al., 2024; Kapoore et al., 2022; Ofoedum et al., 2024). Metabolites are formed by different types of processes such as fermentation, with types of batch, fed-batch and continuous, and biotransformation. Also, their production involves various metabolic pathways that rely on different carbon and nitrogen sources (Srivastava et al., 2024).

Microbial metabolites are typically classified into two major classes which are primary and secondary metabolites. Primary metabolites, also known as central metabolites, directly contribute to the vital activities of microorganisms such as growth, development, and reproduction. These metabolites, which perform a physiological function and/or a fundamental role in the microorganism, are nearly found in all types of microorganisms. Proteins, esters, polysaccharides, nucleic acids, amino acids, enzymes, coenzymes, alcohols (ethanol, etc*.*) and organic acids (citric, acetic, lactic acids, etc*.*) can be given as examples of primary metabolites (Altaf-Ul-Amin et al., 2018; Dwivedi et al., 2019; Hassan et al., 2019; Ofoedum et al., 2024; Silva et al., 2019; Srivastava et al., 2024). Contrary to primary metabolites, secondary metabolites do not directly involve in vital activities of microorganisms and are characterized as low-molecular-mass organic compounds. In general, they are synthesized during the late exponential phase or under nutrient-limited conditions, with their production largely regulated by growth parameters, particularly the composition of the fermentation medium. Antibiotics constitute the most prevalent example of secondary metabolites, followed by toxins, pigments, hormones, enzymes inhibitors, organic acids (γ-aminobutyric acid, citrinin, etc*.*), therapeutic agents, aromatic compounds, as well as growth booster found in both plants and animals (Chen and Wang, 2017; Nagarajan et al., 2022; Netzker et al., 2015; Nigam and Singh, 2014; Salvatore et al., 2025; Tallapragada and Dikshit, 2017).

Monoculture, also called pure culture, is a form of culture that contains only one type of microorganism (Ogodo et al., 2022; Yeh and Hsu, 2019). Unlike monoculture, co-culture, also known as mixed culture or microbial consortium, refers to the either spontaneous or synthetic presence of at least two distinct microorganisms or strains together in a well-defined medium, and interacting with one another in various ways (Kapoore et al., 2022; Peng et al., 2023a, b; Rosero-Chasoy et al., 2021; Schmidt, 2021). Additionally, microorganisms coexist and engage within complex communities in nature. Based on this, the synthetic co-culture strategy artificially assembles different microorganisms to mimic this natural organization under controlled laboratory conditions (Liu et al., 2022).

Microbial co-culture has several advantages which monoculture cannot provide. Improved metabolic efficiency and ecosystem functionality can be achieved in co-culture systems due to their high microbial diversity which also lead to promoting engagement of microbial consortium and its environment that enables the utilization of complex or mixed substrates to produce valuable products, resulting in decreased production costs (Guo et al., 2024; Ray and Behera, 2017; Schmidt, 2021). Also, owing to their multifunctionality, durability, and capability to perform complex metabolic activities, co-cultivation provides stronger resistance to various endogenous and exogenous influences and stresses (Guo et al., 2024; Kapoore et al., 2021; Schmidt, 2021; Xu, 2020). Additionally, the intricate interactions between distinct microorganisms in a mixed culture contribute to microbial growth by preventing the imbalance of allocation of resources, while also promoting metabolite production by providing compatible coworking of metabolic pathways (Guo et al., 2024; Schmidt, 2021). A notable advantage is the prevention of unfavorable metabolites, as inhibitory intermediates or byproducts can be metabolized by specific species within the co-culture system (Peng et al., 2023a, b; Schmidt, 2021). Moreover, by co-cultivation, metabolic burden on microorganisms can be decreased and more consistent process can be achieved through the elimination of unwanted microorganisms (Guo et al., 2024; Peng et al., 2023a, b; Schmidt, 2021).

Utilizing co-cultures holds great promise for improving various industrial applications. In the food industry, the optimization of production processes, valorization of food waste, increase in favorable compounds such as organic acids, ethanol, esters, polyphenols, flavonoids, and polysaccharides, as well as the inhibition of the growth of harmful microorganism and enhancement of sensory properties of food products, can be achieved through the implementation of co-culture systems (Bai et al., 2023; Du et al., 2020; Erdemir-Tıraş and Kalkan-Yıldırım, 2021; Hu et al., 2021; Hua et al., 2022; Li et al., 2025; Ma et al., 2024; Peng et al., 2023a, b; Smid and Lacroix, 2013; Zhang et al., 2024; Zhao et al., 2025a, b). Co-cultivation of microorganisms also makes ways for finding out the new therapeutic natural compounds and enhancing antibiotic production which can contribute to the improvement of the drug industry (Latif et al., 2024; Ueda and Beppu, 2016; Zhuang and Zhang, 2021). Additionally, the application of co-cultivation is used for increasing productivity of bioremediation processes such as wastewater treatment and enhancing or optimizing the production of biofuels such as biodiesel and biohydrogen (Das et al., 2022; Guo et al., 2024; Kapoore et al., 2022; Li et al., 2021; Ori et al., 2024; Pachapur et al., 2015).

Even though co-cultures often exist in food production either naturally or synthetically, studies investigating the background mechanism of metabolite activities of microbial consortium in foods are still inadequate. The purpose of this paper is to present a comprehensive review about the enhancement of metabolites through co-culture approaches, including microbial interactions, modular co-culture and communications within microbial consortia, with a focus on food. Furthermore, the associated challenges and future directions regarding co-cultivation approaches were discussed.

## Increasing metabolites in foods by utilizing co-culture

Production of metabolites in food products can be stimulated by co-cultivation of microorganisms with different approaches such as microbial interactions, modular co-culture, and communications in microbial consortia.

### Metabolite enhancement by microbial interactions

**Fig. 1:**
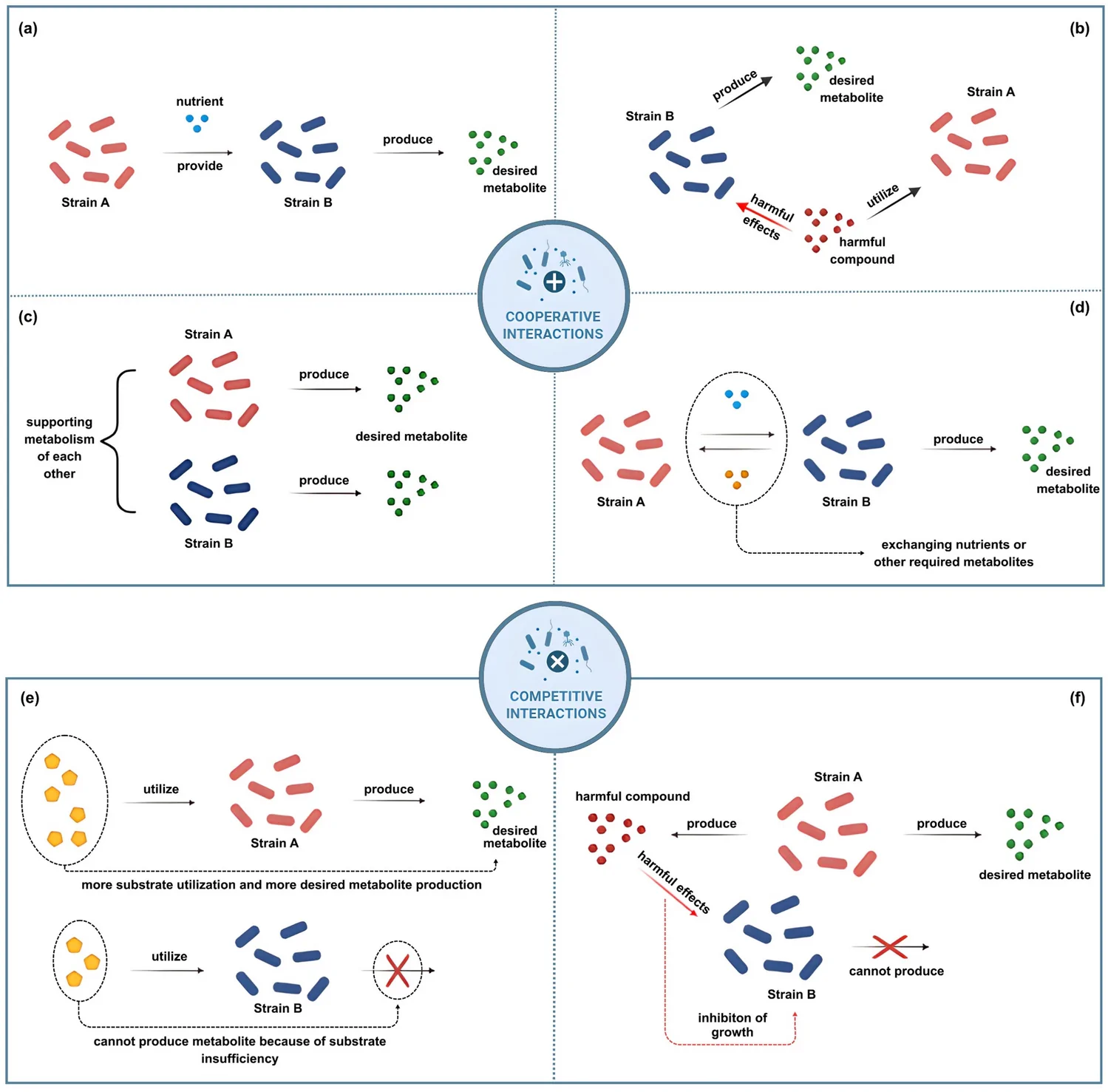
Schematic illustration of microbial interactions. **(A)** Nutrient provision. **(B)** Eliminating harmful compounds. **(C)** Synergistic interactions. **(D)** Cross-feeding. **(E)** Exploitation. **(F)** Interference

The microbial interactions in microbial consortia of food systems significantly influence organoleptic properties, functional attributes, storage durations, and microbial safety of fermented foods (Xin and Qiao, 2025). The interactions between microorganisms within microbial consortia, which can be either cooperative (positive) or competitive (negative), can lead to enhanced metabolite production (Che and Men, 2019; Guo et al., 2024). In Fig. 1, types of microbial interactions were illustrated. Also, microbial interactions in various foods were represented in Table 1.

**Table 1:** Applications of microbial interactions to increase metabolites in foods

Microorganisms	Food product	Type	Metabolites	Quantitative effects	Main outcomes	Refs.
*Limosilactobacillus fermentum* + *Zygosaccharomyces rouxii*	Soy souce	Nutrient provision	HEMF^1^ HDMF^2^	Relative concentration: • Co-culture: 3.21 ± 0.15 *Z. rouxii*: 2.93 ± 0.03 • Co-culture: 0.68 ± 0.04 *Z. rouxii*: 0.39 ± 0.02	• Lactic acid produced by *L. fermentum* enhanced the production of HEMF, and HDMF by *Z. rouxii*	Feng et al., (2024)
		Eliminating harmful compounds	Phenethyl acetate Ethyl acetate	• Co-culture: 1.00 ± 0.27 *Z. rouxii*: 0.05 ± 0.02 • Co-culture: 40.89 ± 1.45 *Z. rouxii*: 40.50 ± 0.78	• *L. fermentum* enhanced metabolite production by reducing the level of acetic acid, which exerts an inhibitory effect on *Z. rouxii*	
*Lactobacillus plantarum* LPBF35 + *Pichia fermentans* YC5.2	Cocoa beans	Synergy	Primary: Lactic acid Acetic acid Secondary: Benzaldehyde Ethyl acetate	Concentration (mg/g): • Co-culture: 0.95 ± 0.03 LPBF35: 0.36 ± 0.03 • Co-culture: 5.24 ± 0.06 LPBF35: 1.39 ± 0.26 Peak area: • Co-culture: 3.35 ± 0.27 LPBF35: 2.24 ± 0.45 • Co-culture: 2.34 ± 0.12 LPBF35: 0.58 ± 0.03	• The co-inoculation resulted in a synergistic interaction, leading to an increased production of both primary and secondary metabolites	Viesser et al., (2021)
*Lachancea thermotolerans* 101 + *Saccharomyces cerevisiae* EC1118	Wine	Synergy	2-Phenylethanol Glycerol	Content: • Co-culture: 31.62 ± 1.28 mg/L 101: 18.93 ± 2.72 mg/L EC1118: 22.23 ± 1.70 mg/L • Co-culture: 7.19 ± 0.05 g/L 101: 7.16 ± 0.25 g/L EC1118: 7.04 ± 0.48 g/L	• Synergistic interactions in co-culture system led to the enhancement of 2-phenylethanol and glycerol production compared to monocultures	Gobbi et al., (2013)
*Lacticaseibacillus rhamnosus* 1473 + *Lactobacillus acidophilus* LMG 8151 + *Pediococcus acidilactici* 3992	Okara	Synergy	*p*-Hydroxy-phenyllacticacid Phenyllacticacid Indole-3-lactic acid Daidzein Genistein	Concentrations (µg/g): • Co-culture: 133.95 ± 4.42 3992: 37.98 ± 4.17 1473: 30.82 ± 1.93 8151: 48.54 ± 3.58 • Co-culture: 191.79 ± 15.05 3992: 48.83 ± 2.81 1473: 32.58 ± 2.40 8151: 96.24 ± 9.10 • Co-culture: 84.91 ± 4.89 3992: 19.77 ± 1.45 1473: 18.65 ± 0.57 8151: 30.65 ± 1.60 • Co-culture: 520.49 ± 27.12 3992: 358.62 ± 5.52 1473: 500.77 ± 20.47 8151: 277.41 ± 25.50 • Co-culture: 532.60 ± 16.61 3992: 303.28 ± 7.72 1473: 494.79 ± 15.67 8151: 376.37 ± 22.62	• The synergistic effect resulting from the co-cultivation of the three microorganisms led to an increased metabolite production compared to their individual cultivation	Saadoun et al., (2021)
*Saccharomyces cerevisiae* Y3401 + *Wickerhamomyces anomalus* Y3604	Baijiu	Synergy	Ethyl acetate β-phenethyl alcohol Phenethyl acetate	Yield (g/L): • Co-culture: 2.40 ± 0.11 Y3604: 1.58 ± 0.05 • Co-culture: 5.23 ± 0.04 Y3401: 1.90 ± 0.07 Y3604: 0.72 ± 0.13 • Co-culture: 3.65 ± 0.26 Y3401: 0.22 ± 0.04 Y3604: 1.22 ± 0.17	• The synergistic fermentation achieved through the use of two distinct yeast species resulted in enhanced production of flavor-related compounds	Fan et al., (2019)
*Lactobacillus reuteri* + yeast (isolated from Jiu Qu)	Cereal-based probiotic foods	Nutrient provision	Lactic acid Ethanol	Changes: • Co-culture: 1.40 g/L Monoculture:1.19 g/L • Enhancement: 98.4%	• *L. reuteri* benefits from yeast by consuming growth promoting nutrients produced by yeast which resulted in enhanced lactic acid and ethanol production	Kedia et al., (2007)
*Clostridium tyrobutyricum* DB041 + *Saccharomyces cerevisiae* YS219 + LAB (*Pediococcus, Lactobacillus, Weissella,* and *Lactococcus*)	Baijiu	Eliminating harmful compounds	Ethyl butyrate Ethyl hexanoate Ethyl pentanoate Isoamyl butyrate Ethyl octanoate	Contents (μg/kg): • Co-culture: 1046.667 ± 57.570 DB041: 519.333 ± 32.655 YS219: 608.236 ± 32.588 • Co-culture: 6255.695 ± 918.558 DB041: 4521 ± 569.221 YS219: 2419.525 ± 99.799 • Co-culture: 335.141 ± 173.952 DB041: 137.568 ± 27.882 YS219: 71.693 ± 2.143 • Co-culture: 899.391 ± 384.482 DB041: 358.265 ± 165.320 YS219: 423.664 ± 270.414 • Co-culture: 310.349 ± 77.440 DB041: 13.467 ± 3.325 YS219: 399.695 ± 137.149	• The stress on microorganisms caused by the accretion of ethanol, acetic and lactic acid was reduced by supplying acidity balance with the interaction of *S. cerevisiae* YS219 and *C. tyrobutyricum* DB041, resulting in enhanced microbial growth and primary flavor compounds production	Qiu et al., (2024)
*Debaryomyces hansenii* D1 + *Staphylococcus xylosus* S1 + *Pediococcus pentosaceus* PP1	Fermented sausages	Cross-feeding	2-Methyl-butanal 3-Methyl-butanal 3-Methyl-butanol	-^3^	• The proliferation of *S. xylosus* S1 and P*. pentosaceus* PP1 was enhanced by amino acids provided by *D. hansenii* D1 • The consumption of α-keto acids supplied by *D. hansenii* D1 enhanced the bacterial production of branched-chain aldehydes • *D. hansenii* D1 simultaneously synthesized branched-chain alcohols by utilizing branched-chain aldehydes provided by both bacteria	Liu et al., (2023)
Commercial starter culture YO-MIX 300 (*Lactobacillus bulgaricus* CL + *Streptococcus thermophilus* CS) + *Lactococcus lactis* NGD8	Fermented milk	Nutrient provision	Hippuric acid Methylmalonic acid Undecanoic acid Ethyl acetate Dimethyl ether Hexanoic acid Octanoic acid Lactic acid *N*-Methyl-D-aspartic acid Phenyl ethanol 1-Pentanol Acetic acid Pentyl ester 2-Oxoglutaric acid Ethanol	-	• Commercial starter culture provided amino acids to *L. lactis* NGD8, resulting in enhancement of organic acids, esters and bioactive compounds which led to exhibition of unique flavor attributes	Guo et al., (2025)
*Tetragenococcus halophilus* 9477 + *Zygosaccharomyces rouxii* 1682	Soy souce	Competition	2-Methyl-1-propanol 2,5-Dimethylpyrazine Trimethylpyrazine	Relative peak area: • Co-culture: 0.08 ± 0.00 9477: 0.01 ± 0.00 1682: 0.06 ± 0.01 • Co-culture: 2.00 ± 0.38 9477: 0.17 ± 0.02 1682: 0.05 ± 0.01 • Co-culture: 0.15 ± 0.02 9477: 0.05 ± 0.01 1682: 0.05 ± 0.01	• Competitive interactions between both microorganisms led to an increment in pH (7.31) which favored the growth of *T. halophilus* while limiting the proliferation of *Z. rouxii* • *Z. rouxii* showed enhanced alcohol and ester production in this condition, resulting in richer aromatic composition	Devanthi et al., (2018)
*Enterococcus faecalis* 41FL1 + *Staphylococcus aureus* ATCC	Fresh cheese	Competition	Lactic acid Acetoin	Concentration (g/kg): • Co-culture: 31.61 ± 2.61 ATCC: 19.88 ± 4.89 • Co-culture: 2.88 ± 0.27 41FL1: 1.94 ± 0.16 ATCC: 1.02 ± 0.59	• *E. faecalis* 41FL1 suppressed proliferation of *S. aureus* ATCC which brought about an increase in acetoin and lactic acid • The enhancement of acetoin was associated with a mechanism utilized by *S. aureus* ATCC to protect itself from environmental stress factors caused by *E. faecalis* 41FL1	Viçosa et al., (2019)
*Clostridium kluyveri* H068 + *Methanogen* 166	Chinese strong flavor liquor	Eliminating harmful compounds	Caproic acid	• Co-culture: 410.21 mg/100 mL Monoculture: 253.69 mg/100 mL • Enhancement: 61.7%	• Removal of inhibitory hydrogen by *Methanogen* 166 enhanced caproic acid synthesis in *C. kluyveri* H068	Yan and Dong, (2018)
*Zygosaccharomyces rouxii* + *Wickerhamiella versatilis*	Soy souce	Cross-feeding	• HEMF • HDMF • 4-ethyl-2-methoxyphenol • Phenylethanol • 3-methyl-1-butanol • 2-methyl-1-butanol	Fold enhancement: • 1.67 • 1.07 • 7.05 • 1.01 • 1.15 • 1.35	• *Z. rouxii* and *W. versatilis* cross-fed each other with the metabolites they produced which led to an increased production of key aromatic compounds in both yeast species	Wang et al., (2024a, b)

^1^HEMF: 4-hydroxy-2(or 5)-ethyl-5(or 2)-methyl-3(2*H*)-furanone, ^2^HDMF: 4-hydroxy-2,5-dimethyl-3(2*H*)-furanone, ^3^-: not detected

### Cooperative interactions

Cooperative interactions among microorganisms, such as nutrient provision (commensalism), eliminating harmful compound, synergy, and cross-feeding (mutualism), promote a harmonious and resilient presence within co-culture systems, thereby enhancing survival capabilities of microorganisms and improving the production of natural metabolites (Guo et al., 2024; Schmidt, 2021).

Nutrient provision, a type of commensalism, is an interaction where one microorganism supplies growth promoting materials to another microorganism in co-culture system while it remains unaffected in positive or negative manner (Che and Men, 2019; Guo et al., 2024; Jin et al., 2024). Eliminating harmful compounds is another type of cooperative interaction where one microbial strain can enhance the productivity of another by mitigating toxic environmental factors with consumption or migration of toxic metabolites, potentially transforming these harmful compounds into beneficial nutrients that support growth and increase metabolite secretion (Guo et al., 2024). Feng et al. (2024) investigated how interaction of *Limosilactobacillus fermentum* and *Zygosaccharomyces rouxii* in co-culture system affected flavor quality of soy sauce. According to results, lactic acid synthesized by *L. fermentum* was found to stimulate the biosynthesis of 4-hydroxy-2(or 5)-ethyl-5(or 2)-methyl-3(2*H*)-furanone (HEMF), and 4-hydroxy-2,5-dimethyl-3(2*H*)-furanone (HDMF) in *Z. rouxii*. In addition to this commensal interaction, it was found that *L. fermentum* enhanced metabolite production by reducing acetic acid, which has an inhibitory effect on *Z. rouxii*.

Another cooperative interaction called synergistic interactions provides enhanced adaptability and performance of the cooperating microorganisms compared to their performance in individual cultures which leads to efficient secretion of metabolites in a common cultivation setting (Guo et al., 2024; Sarsan et al., 2021). In a study, it was found that co-culturing of the three microorganisms, which are *Lacticaseibacillus rhamnosus* 1473, *Lactobacillus acidophilus* LMG and *Pediococcus acidilactici* 3992, resulted in a synergistic interaction that enhanced production of metabolites such as *p*-hydroxy-phenyllactic acid, phenyllactic acid, indole-3-lactic acid, daidzein and genistein in okara, relative to when each strain was cultured individually (Saadoun et al., 2021).

Within microbial communities, cross-feeding represents a mutualistic interaction characterized by the reciprocal exchange of metabolites, which confers benefits upon all participating microorganisms (Che and Men, 2019; Mayo et al., 2021; Xin and Qiao, 2025). The phenomenon of cross-feeding is contingent upon the translocation and subsequent uptake of specific compounds across distinct species or genotypes of microorganisms, resulting in altered fitness outcomes for both the donor and recipient (Liang et al., 2022), which leads to improved nutrient intake, thereby governing metabolic activities and promoting metabolite synthesis (Guo et al., 2024). Liu et al. (2023) aimed to examine how interactions between three microorganisms, which are *Debaryomyces hansenii* D1, *Staphylococcus xylosus* S1 and *Pediococcus pentosaceus* PP1, affected the production of branched-chain aldehydes which is responsible for flavor of fermented sausages. The results showed that the enhancement of *P. pentosaceus* PP1 and *S. xylosus* S1 proliferation was performed through eight different amino acids such as alanine, aspartate, glutamate, glutamine, glycine, phenylalanine, serine, and threonine provided by *D. hansenii* D1 which also increased the synthesis of branched-chain aldehydes by delivering α-keto acids to both bacteria in cross-feeding manner. In the same study, it was noted that branched-chain aldehydes, which were produced by both bacteria and *D. hansenii* D1, were utilized by *D. hansenii* D1 to convert them into branched-chain alcohols, resulting in an increment of these compounds in co-cultured samples.

### Competitive interactions

The second type of microbial interactions is competitive interactions where microorganisms cohabiting a shared environment often compete for limited nutrients and environmental resources (exploitation) or sometimes secrete toxic chemicals to inhibit or eliminate rivals (interference) which lead to increase the production of existing metabolites or even stimulate the synthesis of novel ones (García et al., 2019; Ghoul and Mitri, 2016; Guo et al., 2024). Viçosa et al. (2019) demonstrated that when *Enterococcus faecalis* 41FL1 was co-cultured with *Staphylococcus aureus* ATCC in a fresh cheese model for 21 days of storage (fermentation continued), the growth of *S. aureus* ATCC was inhibited by *E. faecalis* 41FL1 which resulted in increased lactic acid compared to monoculture of *S. aureus* ATCC. Moreover, acetoin content was elevated in co-culture system relative to both single cultures. This increment of acetoin in co-cultured cheese was described as a coping mechanism employed by *S. aureus* ATCC to handle the environmental stress factors such as low pH and redox potential caused by *E. faecalis* 41FL1, through adjusting carbon flux to generate less acidic byproducts.

## Metabolite enhancement by modular co-culture

**Fig. 2:**
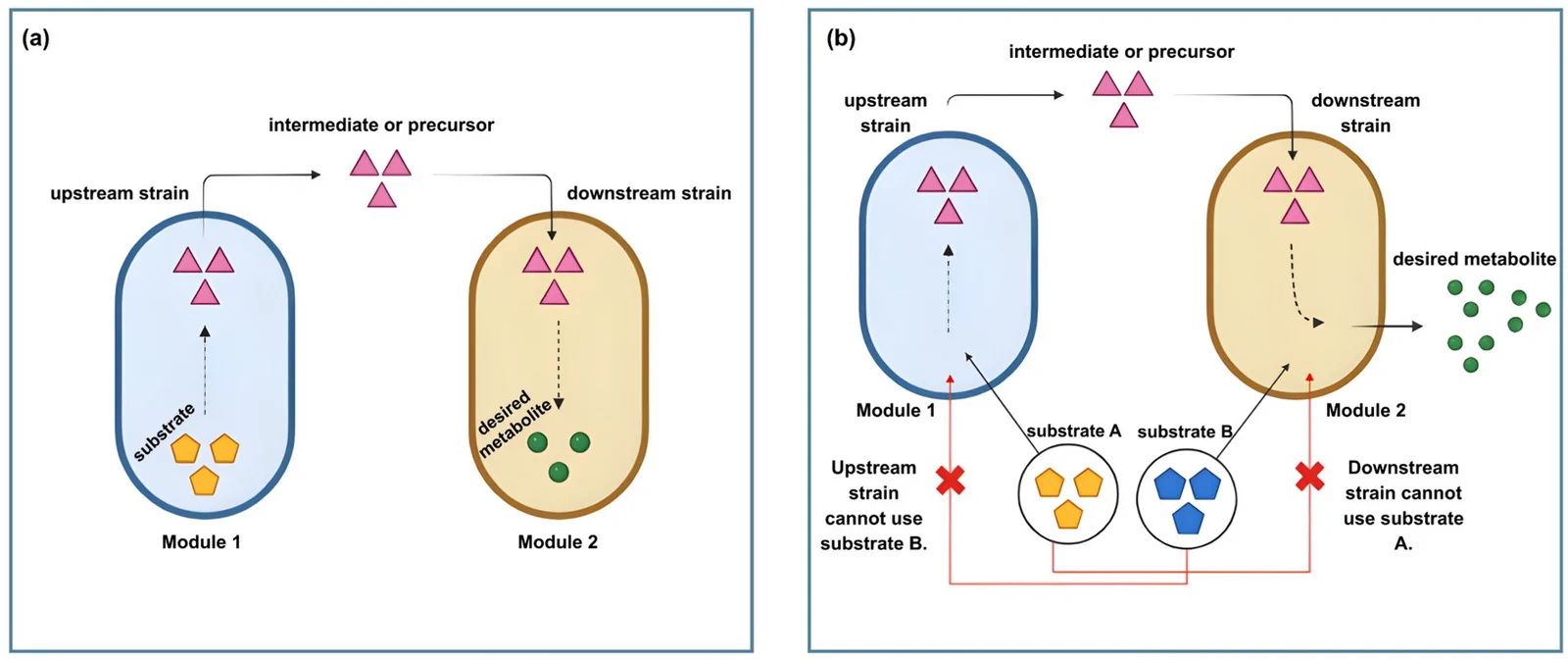
Schematic illustration of modular co-culture. **(A)** Division of labor: producing and transporting intermediates—Precursor directed synthesis: producing and transporting precursors. **(B)** Improve substrate utilization: modifying strains to make them use only one specific substrate

Another metabolite-enhancing strategy is modular co-culture, which is based on the allocation of the entire biosynthetic pathway to distinct modules, each carried out by a different microorganism within the co-culture system (Wang et al., 2020; Zhao et al., 2023). Modular co-culture offers several advantages, including easier management of metabolic flux by adjusting cell population ratios, reduced metabolic burden and feedback inhibition, and a broader capacity to utilize diverse substrates and secrete miscellaneous or unattainable metabolites more efficiently than monocultures (Zhao et al., 2023). All these advantages can be supplied by division of labor, which is the core mechanism of modular co-culture strategy, where upstream and downstream modules are constructed to divide complex biosynthetic pathway (Mittermeier et al., 2023) for efficient production of targeted metabolite (Lin et al., 2024). In this mechanism, microorganisms in upstream modules produce intermediates which are later utilized by microorganisms in downstream modules to synthesize desired metabolites (Jones and Wang, 2018; Mittermeier et al., 2023). Additionally, division of labor has specialized forms, which are known as precursor-directed metabolite synthesis and improving substrate utilization. Illustration of modular co-culture types, and modular co-culture applications to increase food related metabolites were given in Fig. 2 and Table 2, respectively.

**Table 2:** Applications of modular co-culture to increase food related metabolites

Microorganisms	Type	Metabolites	Quantitative effects	Main outcomes	Refs.
*E. coli* DPAK5 + *E.coli* ALA11-8 + *E.coli* DPAK6	Precursor Directed Biosynthesis	D-pantothenic acid (D-PA)	Titer and productivity at 72 h in a 5 L fermenter: • Single culture (DPAK5): 52.8 g/L and 0.73 g/L/h • Dual-strain co-culture (DPAK5 and ALA11-8): 25.4 g/L and 0.35 g/L/h • Triple-strain co-culture: 41.9 g/L and 0.58 g/L/h	• Higher D-PA production was observed in single-strain fermentation, with a requirement of external β-alanine supplementation • By using dual- and triple-strain systems high levels of D-PA production were achieved without the addition of exogenous β-alanine • D-PA production of triple- strain co-culture was higher than dual-strain co-culture	Qin et al., (2025)
*Bacillus velezensis* JNB3 + *Saccharomyces cerevisiae* JNY1 + *Lentilactobacillus buchneri* JNL1	Precursor Directed Biosynthesis	3-(methylthio)-1-propanol	Yield: • Co-culture: 446.14 ± 39.83 µg/L • Single culture (*S. cerevisiae* JNY1): 173.30 ± 12.65 µg/L Enhancement: approximately 157.4%	• In this modular co-culture system, 3-(methylthio)-1-propanol content was increased by approximately 157.4% relative to single culture	Du et al., (2021)
*E. coli* strain (HSEC0916) + *E. coli* strain (HSEC1017)	Improve Substrate Utilization	Isobutyl Butyrate	At 1:4 (HSEC0916: HSEC1017) ratio: • Butyrate selectivity: 46% mol/mol • Titer: 133 ± 14 mg/L	• The 1:4 inoculation ratio resulted in higher isobutyl butyrate selectivity and titer relative to other tested ratios (2:1, 1:1, 1:2, 1:9) and the monoculture	Seo et al., (2024)
*Saccharomyces cerevisiae* RLAs1654 + *Saccharomyces cerevisiae* RLAs1642 + *Saccharomyces cerevisiae* RLAs1643	Precursor Directed Biosynthesis	Raspberry Ketone (RK)	Titer in SC^1^ medium: • CL_RK2^2^: 11.7 mg/L (14% increment) • RLAs1643 10.3 mg/L • CL_RK3^3^: 10.0 mg/L Titer in SM^4^ medium: • CL_RK3: 13.4 mg/L • RLAs1642: 8.2 mg/L	• In SC medium and at a 1:100 ratio, RK production of CL_RK2 was higher than RLAs1643 • In SM medium and at a 1:20:1 ratio, RK level of CL_RK3 was higher than RLAs1642 • In SC medium, the RK titer of CL_RK3 was comparable to RLAs1643 • Compared to the highest RK titers of CL_RK1^5^ and CL_RK2, CL_RK3 exhibited 63.8% and 37.5% increases in RK production, respectively	Peng et al., (2023a, b)
*Yarrowia lipolytica* (H222-cart) (Wild Type-WT) + *Yarrowia lipolytica* (ΔHXT (evl)-cart)	Improve substrate utilization	β-carotene	Titer in synthetic minimal media: • Co-culture: 998 mg/L • H222-cart: 427 mg/L • ΔHXT (evl)-cart: 198 mg/L Titer in lignocellulosic hydrolysate media: • Co-culture: 4101 mg/L • H222-cart: 2009 mg/L	• In synthetic minimal media, after 10 days, the co-culture outperformed the single culture of H222-cart and ΔHXT (evl) in β-carotene production • In lignocellulosic hydrolysate media and at day 10, the co-culture demonstrated higher β-carotene production compared to the single culture of H222-cart • β-carotene production in lignocellulosic hydrolysate media was higher than in synthetic minimal media	Rafieenia et al., (2025)
*Wickerhamomyces anomalus* + *Pantoea* sp*.* + *Bacillus altitudinis*	Precursor Directed Biosynthesis	4-ethyl guaiacol	Yield: • Tri-culture: 37.41 µg/L • Monocultures: < 0.001 mg/L • Bi-cultures: 9.08 µg/L • Quadri-cultures: 8.13 µg/L • Penta-cultures: 12.07 µg/L	• The tri-cultures consisting of *W. anomalus*, *Pantoea* sp. and *B. altitudinis* at 1:1:1 ratio showed the highest 4-ethyl guaiacol yield compared to monocultures, bi-cultures, quadri-cultures and penta-cultures	Yan et al., (2024)
*E. coli* (SA-Ec4) + *Candida glycerinogenes* (CA-Cg27)	Precursor Directed Biosynthesis	Caffeic Acid	In optimized conditions: • Co-culture: 871.9 mg/L • CA-Cg27: 346.5 mg/L • Enhancement: 151.6% In 5 L bioreactor: • Co-culture (under mixed sugar conditions: 2311.6 mg/L • Enhancement: 17.2-fold • Co-culture (containing sugarcane bagasse hydrolysate): 1943.2 mg/L • Enhancement: 14.6-fold CA-Cg3^6^: 134.0 mg/L	• The optimized co-culture system demonstrated caffeic acid content of co-culture at a 1:10 (SA-Ec4:CA-Cg27) ratio was higher than CA-Cg27 • In both sugar conditions, caffeic acid was higher than single culture of CA-Cg3 • In a 5 L bioreactor, the caffeic acid titers of co-culture systems containing mixed sugar and sugarcane bagasse hydrolysate increased 2.7 and 2.2 times relative to the shake flask scale	Wang et al., (2024a, b)
*Saccharomyces cerevisiae* sNAR5 + *Saccharomyces cerevisiae* sDPD3 or *Saccharomyces cerevisiae* sDPD4 or *Saccharomyces cerevisiae* sDPD5	Precursor Directed Biosynthesis	Delphinidin	Titers after the 72-h fermentation: • sNAR5 + sDPD3: 15.8 mg/L • sNAR5 + sDPD4: 18.8 mg/L • sNAR5 + sDPD5: 21.5 mg/L Titer-Enhancement in optimized conditions: • sNAR5 + sDPD5 (7:3): 26.1 mg/L-approximately 2 times sDPD2^7^: 13.3 mg/L	• The delphinidin levels of all co-culture type outperformed the single culture of sDPD2 • The optimized system was constructed by co-culturing of sNAR5 and sDPD5 (7:3), resulting in nearly twofold increment in delphinidin relative to sDPD2 • This system was implemented to produce different flavonoids such as kaempferol, quercetin, myricetin, pelargonidin and cyanidin by co-culturing sNAR5 with different engineered strains	Du et al., (2020)

^1^SC: synthetic complete dextrose, ^2^CL_RK2: *S. cerevisiae* RLAs1654 + *S. cerevisiae* RLAs1643, ^3^CL_RK3: *S. cerevisiae* RLAs1654 + *S. cerevisiae* RLAs1642 + *S. cerevisiae* RLAs1643, ^4^SM: synthetic minimal, ^5^CL_RK1: *S. cerevisiae* RLAs1654 + *S. cerevisiae* RLAs1642, ^6^CA-Cg3: an engineered *C. glycerinogenes* strain producing caffeic acid, which is resistant to feedback inhibition (harboring the ARO4^K229L^ and ARO7^G141S^ mutations), ^7^sDPD2: *S. cerevisiae* strain harboring the entire delphinidin pathway, with gene expression enhanced by strong promoters

In precursor directed synthesis, precursors are produced by upstream microorganisms as intermediates to provide them for downstream microorganisms, while in substrate utilization improvement, the carbon sources of mixed substrate are apportioned among the microorganisms in each module to inhibit substrate competition, respectively (Guo et al., 2024; Rafieenia et al., 2022). Du et al. (2021) set up a co-culture system involving modular division of labor of the biosynthetic pathway of 3-(methylthio)-1-propanol production to increase its production yield. In this system, the biosynthetic pathway of 3-(methylthio)-1-propanol was divided into three modules where *Bacillus velezensis* JNB3, *Saccharomyces cerevisiae* JNY1 and *Lentilactobacillus buchneri* JNL1 were assigned to module 1, 2 and 3 which served as methionine (precursor) synthesizer, 3-(methylthio)-1-propanol (product) synthesizer and methyl-cycle enhancer modules, respectively. According to results, the biosynthesis of 3-(methylthio)-1-propanol, also known as key sulfur compound, characterized by a cauliflower-like and cooked vegetable aroma in Baijiu, was remarkably enhanced by about 157.4% with the maximum production yield of 446.14 ± 39.83 µg/L compared to single culture of *S. cerevisiae* JNY1 (173.30 ± 12.65 µg/L). In another study, modular co-culture system containing two specialized *Escherichia coli* in substrate utilization was constructed to address challenges in de novo biosynthesis of isobutyl butyrate, which is used in ice cream and confections for fruity flavors (National Center for Biotechnology Information, 2025), from mixture of glucose and xylose by monoculture. Firstly, an *E. coli* strain (HSEC0916) was engineered to consume glucose as a single sugar source which utilizes endogenous carbon catabolite repression (CCR) regulatory system to initiate glucose uptake. On the other hand, a xylose-preferring *E. coli* strain (HSEC1017) that prioritizes xylose uptake over glucose was constructed, effectively suppressing CCR. Following strain engineering, the isobutyl butyrate biosynthetic pathway was divided into isobutanol, butyl-CoA, and ester condensation modules, where HSEC0916 was assigned to isobutanol module, while HSEC1017 was assigned to butyl-CoA and ester condensation modules. According to the results, in HSEC0916/HSEC1017 inoculation ratio of 1:4, it was found that the isobutyl butyrate selectivity (46% (mol/mol)) and titer (133 ± 14 mg/L) was higher than other inoculation ratios (2:1, 1:1, 1:2, 1:9) and monoculture (Seo et al., 2024).

## Metabolite enhancement by microbial communication

**Fig. 3:**
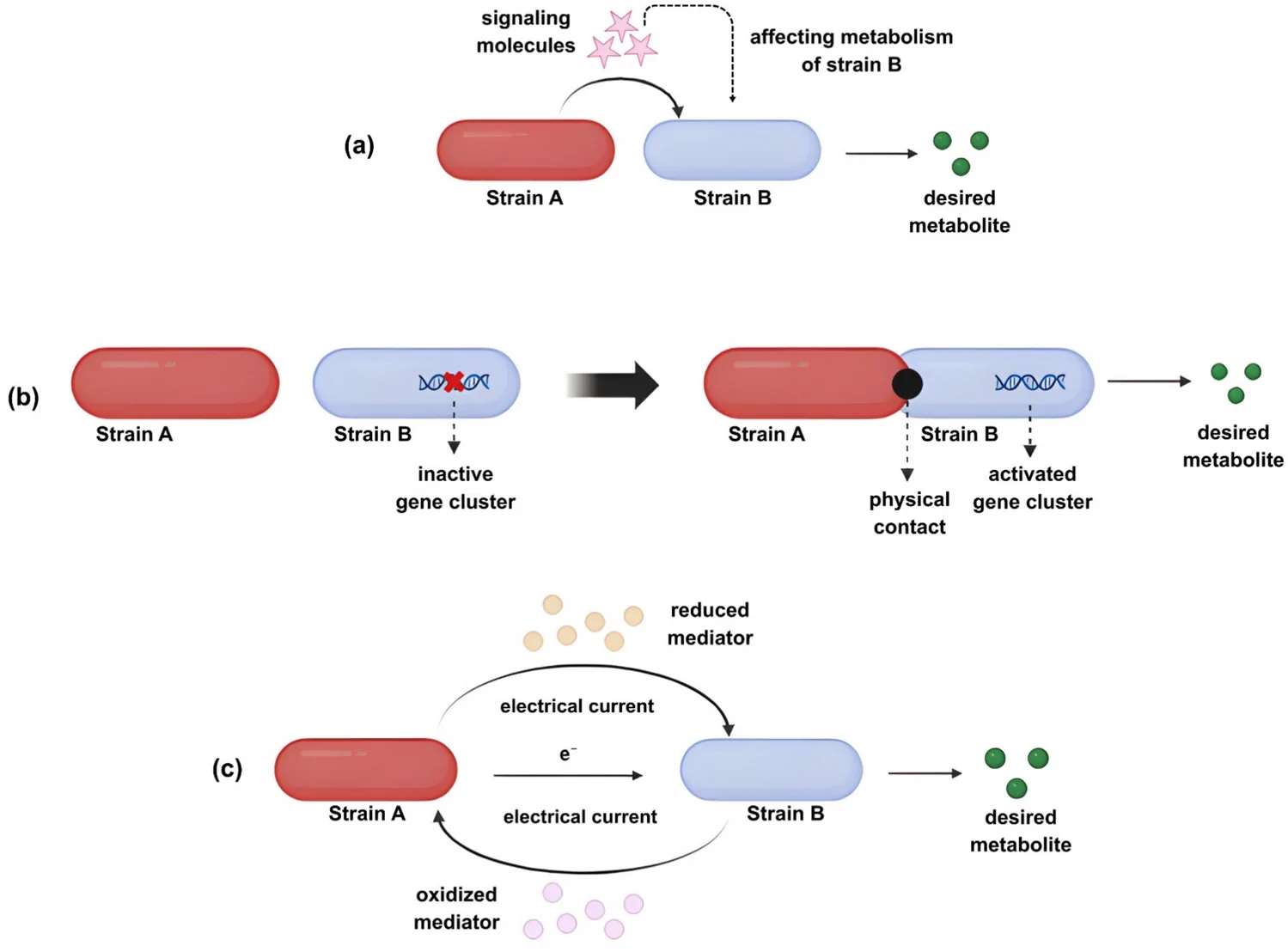
Schematic illustration of microbial communication. **(A)** Quorum sensing. **(B)** Physical contact. **(C)** Electrical signaling

Microbial communication is another approach to increase microbial metabolites by organizing microbial activities within a consortium. As a part of sociomicrobiology, it involves the exchange of signaling molecules between microorganisms of the same or different species to control gene expression and synchronize collective behavior (Pan et al., 2023). Microbial communication occurs in three main forms, which are quorum sensing, physical contact, and electrical signaling. Microbial communication types were illustrated in Fig. 3, and effect of microbial communication on enhancing food related metabolites were shown in Table 3.

**Table 3:** Applications of microbial communication to increase food related metabolites

Microorganisms	Type	Metabolites	Quantitative effects	Main outcomes	Refs.
*Enterococcus faecium* AB157 + *Saccharomyces cerevisiae* SC125	Quorum Sensing	GABA	Yield (g/L): • Co-culture: 6.57 • AB157 + SC1125 (CFS^1^): 4.61 • AB157: 3.68 • SC125: 0.13	• The addition of CFS from *S. cerevisiae* SC125 to *E. faecium* AB157 increased AIP synthesis and sensor HK activity in *E. faecium* AB157, thereby leading to an enhancement of GABA production	Tan et al., 2024a
*Lactiplantibacillus plantarum* BC112 (CFS) + *Enterococcus faecium* AB157	Quorum Sensing	GABA	Content (g/L): • Co-culture: 1.62–3.42 (3–9% CFS) • AB157: 1.48 Enhancement: up to 4.6-fold	• CFS application to AB157 induced *luxS* gene expression, which in turn increased AI-2 activity in *E. faecium* AB157, thereby enhancing GABA production relative to non-CFS sample • Total GABA increase was influenced not only by QS but also by enhanced GAD^2^ activity, gene expression, optimized pH, and CFS-derived glutamic acid	Tan et al., 2024b
*Enterococcus faecium* KL66 + *Kluyveromyces marxianus* KY7 + *Acetobacter fabarum* KA26	Quorum Sensing	Kefir grain formation through biofilm	Biofilm Quantity (OD_560_): • Co-culture: 3.83 ± 0.094 • KL66: 0.10 ± 0.0082 • KY7: 0.30 ± 0.024 • KA26: 0.16 ± 0.022	• At 4 h, 37 °C, and pH 7.5, AI-2 synthesis and biofilm development reached their optimum by achieving the highest AI-2 fluorescence intensity (6.45 ± 0.27) and biofilm level (3.83 ± 0.094), outperforming single cultures, double co-cultures, and other triple co-cultures • Strong link between AI-2 and biofilm production existed, due to an increment in biofilm development as AI-2 synthesis increased	Zhang et al., 2025a, b
*Lachancea thermotolerans* IWBT Y1240 (Lt) + commercial *Saccharomyces cerevisiae* strain Lalvin EC1118™ (Sc)	Physical Contact	2-Phenylethanol Propionic acid Butyric acid Hexanoic acid	Major volatile compounds (mg/L): • Co-culture: 74.62 ± 19.75 Sc^3^: 42.62 ± 12.02 Lt^4^: 32.77 ± 4.84 • Co-culture: 2.72 ± 0.05 Sc: 1.23 ± 0.14 Lt: 1.03 ± 0.23 • Co-culture: 0.80 ± 0.01 Sc: 0.61 ± 0.00 Lt: 0.47 ± 0.03 • Co-culture: 0.65 ± 0.01 Sc: 0.51 ± 0.06 Lt: 0.32 ± 0.04	• The physical contact of yeasts in a synthetic grape must medium increased the production of major volatile compounds • The culturability and viability of *L. thermotolerans* were reduced compared to monocultures and non-physical contact co-cultures	Luyt et al., 2021
*Shewanella putrefaciens* CN32 and *Geobacter soli* (Signal routers) + *Rhodopseudomonas palustris* TIE-1 (Bio-actuator)	Electrical Signaling	Lycopene	Increment compared to monoculture: • CN32 + TIE-1: 1.2-fold • Triple co-culture: 1.55-fold	• Iron ion reduction and oxidation were provided by signal routers and a bio-actuator, respectively • Lycopene yields increased in both binary and triple mixed cultures, due to enhanced NADPH/NADH generation and ATP synthesis in *R. palustris* via the redox-based communication system	Chen et al., 2023

^1^CFS: cell-free supernatant, ^2^GAD: Glutamate Decarboxylase, ^3^Sc: commercial *Saccharomyces cerevisiae* strain Lalvin EC1118™, ^4^Lt: *Lachancea thermotolerans* IWBT Y1240

Quorum sensing (QS) is a communication system depending on microbial density, which involves the release of small signaling molecules, known as autoinducers (AIs), outside the cell, followed by being detected via receptor proteins in other microorganisms and triggering coordinated gene expression once a specific cell density is achieved. The most prevalent AIs include various signaling molecules such as *N*-acyl-homoserine lactones (AHLs), autoinducing peptides (AIPs) and cyclic furanone compound (by-product of active methyl cycle) which are utilized by Gram-negative bacteria in the AI-1 system, Gram-positive bacteria and both types of bacteria in AI-2 system, respectively (Abbamondi and Tommonaro, 2022). With these signaling molecules in QS systems, microorganisms can suppress spoilage-related or pathogenic microorganism production, enhance favorable microorganisms and metabolites in food products which results in providing desirable sensory attributes and safety (Guo et al., 2024; Rul and Monnet, 2015). Tan et al. (2024a) investigated whether γ-aminobutyric acid (GABA) content can be increased by co-culturing *Enterococcus faecium* AB157 and *Saccharomyces cerevisiae* SC125, which are obtained from Sichuan Pao Cai. Co-cultivation of *E. faecium* AB157 and *S. cerevisiae* SC125 at 4:1 ratio and 35 °C with 12 g/L monosodium glutamate (MSG) was determined as optimized conditions for production of GABA with the highest value of 6.57 g/L, which was also higher than single culture of *E. faecium* AB157 (3.68 g/L) and *S. cerevisiae* SC125 (0.13 g/L). In this co-cultivation system, both microbial interactions and communications were involved in induced GABA production. Therefore, to specifically assess the effect of the QS on the production of GABA in the co-culture system, cell-free supernatant (CFS) of *S. cerevisiae* SC125 was added to *E. faecium* AB157 and transcriptomic analysis was performed. The results demonstrated that the highest value of GABA with 4.61 g/L was obtained in the sample of *E. faecium* AB157 treated with 12 h-fermented *S. cerevisiae* SC125 CFS. This enhancement of GABA production was associated with a QS mechanism where AIP synthesis of *E. faecium* AB157 was increased by CFS of *S. cerevisiae* SC125 which resulted in increment of sensor histidine kinase (HK) activity in *E. faecium* AB157, thereby enhancing its viability and GABA biosynthesis.

Microbial communication can also take place through physical contact between signaling and detecting species which lead to identification of new metabolites or increment of already available ones by activating silent gene cluster (Denef, 2014; Guo et al., 2024). Luyt et al. (2021) aimed to determine effects of physical contact of *Lachancea thermotolerans* IWBT Y1240 and commercial *Saccharomyces cerevisiae* strain Lalvin EC1118™, which are involved in wine fermentation, on metabolite production along with culturability and viability of yeasts compared to monocultures and non-physical contact co-culture of both yeasts in a synthetic grape must medium. According to results, it was found that culturability and viability of *L. thermotolerans* decreased in the sample of co-culture with physical contact while culturability of *S. cerevisiae* decreased in non-physical contact sample. In metabolite production, higher concentration of major volatile compounds such as 2-phenylethanol (74.62 ± 19.75 mg/L), butyric acid (0.80 ± 0.01 mg/L), hexanoic acid (0.65 ± 0.01 mg/L) and 2-phenylethyl acetate (0.19 ± 0.02 mg/L) were obtained in the sample of co-culture with physical contact compared to other samples. However, it was indicated that there were no remarkable changes in ethanol and acetic acid production among the samples.

Lastly, an increase in metabolites can be achieved through electrical signaling, where electrons are exchanged between electroactive microorganisms in a co-culture system, which results in the formation of an electrical current that enables microbial communication (Guo et al., 2024; Paquete et al., 2022). Chen et al. (2023) developed a redox-based communication system that is initiated by electron transfer and composed of three key parts, which were a signal router, an optical verifier, and a bio-actuator, aimed at controlling and synchronizing microbial metabolism. In this co-culture system, *Shewanella putrefaciens* CN32 and *Geobacter soli* acted as signal routers which were in charge of reducing iron ions from Fe^3+^ to Fe^2+^, while *Rhodopseudomonas palustris* TIE-1 was used as bio-actuator which were responsible for oxidizing Fe^2+^ to Fe^3+^ ions and producing lycopene. Two types of co-culture system, which were binary and triple mixed culture including *S. putrefaciens* – *R. palustris* and *S. putrefaciens* + *G. soli* (dual-band router) – *R. palustris*, respectively, were constructed and activities of microorganisms were examined. According to results, it was obtained that lycopene production yield increased in both binary and triple mixed culture system by 1.12 and 1.55 times, respectively, compared to mixed culture without applying redox communication system. It was stated that this increased lycopene yield was associated with increased NADPH and NADH in *R. palustris* by gaining electrons from redox-based communication system where oxidation and reduction of iron ions occurred, which resulted in increased ATP synthesis and enhanced efficiency of oxidation/reduction activities of microorganisms.

## Challenges and future insights

Alongside its significant benefits, co-culturing also has several important challenges. The culturing of multiple populations in co-culture systems is complex due to the distinct inherent traits of microorganisms, uncertainty of microorganism behaviors towards each other and the difficulty in controlling an increasing number of species or strains (Jawed et al., 2019; Zhao et al., 2023). These dynamic variations of each microorganism in co-culture system make it hard to maintain the stability and robustness of the microbial community (Brenner et al., 2008; Guo et al., 2024; Jones and Wang, 2018; Zhang and Wang, 2016; Zhao et al., 2023). Additionally, these systems face an elevated risk of contamination (Prabhu et al., 2022). Besides these adversities, interactions and communications between microorganisms are still not fully elucidated which complicate construction of mixed culture systems (Che and Men, 2019). Another challenge in co-cultivated systems is difficulty of determination of metabolites and metabolite fluxes with high efficiency and accurateness which is crucial for understanding and determining behaviors of microorganisms towards each other. Also, scaling up the systems containing co-cultures to produce metabolites for adapting to industrial applications is difficult in terms of imbalanced nutrient distribution, fluctuating environmental conditions and ensuring cost–utility balance (Guo et al., 2024; Tan et al., 2019; Zhao et al., 2023). Lastly, regarding the modular co-culture method, the efficient mass transfer of pathway intermediates between upstream and downstream strains of each module is a major bottleneck, which affects the production efficiency of desired metabolites, since biosynthetic molecules such as CoA derivatives and phosphorylated metabolites exhibit low membrane permeability, restricting their function as intercellular metabolic shuttles (Zhang and Wang, 2016; Zhao et al., 2023).

Future studies for enhancement of metabolites via co-cultivation should focus on overcoming current obstacles of co-culture systems and improving these systems to obtain new or increased microbial metabolites. In future, microorganism relationships, their metabolic activities and proliferation behaviors should be comprehended in detail by integrating highly advanced and efficient technologies such as microfluidic instruments, genome editing and screening platforms, as well as implementing affordable gene-chip assays into co-culture systems for constructing more stable and robust co-culture systems to obtain valuable metabolites (Brenner et al., 2008; Che and Men, 2019; Jones and Wang, 2018; Lin et al., 2024). For providing deeper insights into metabolite fluxes in co-culture systems, biosensors (Xu et al., 2020) and state-of-the-art analytical approaches including nano-electrospray ionization, simple targeted quantification, mass spectrum imaging and differential metabolite profiling can be used alongside with classic analytical analysis (Guo et al., 2024). In a study conducted by Zhao et al. (2025a, b), a droplet-based microfluidic platform integrated with dual color *E. coli* biosensors, which provide high-throughput screening, was developed to detect mutant strains of *Saccharopolyspora erythraea* that increase erythromycin production. In this system, green and red fluorescence protein existing in *E. coli* biosensors measured erythromycin and cell density, respectively. On the other hand, a drop-based microfluidic platform enabled the screening of the numerous variants individually and at high speed by enclosing producer and sensor cells in microscopic droplets. The results showed that this system achieved a higher targeted colony rate (91.7%) than single-color screening (83.3%), with enrichment ratios of 99.4 and 44.9, respectively, thereby enabling the successful isolation of mutants that resulted in a 19.6% increase in erythromycin production. In another study, a caffeate‑responsive biosensor, which automatically adjusts strain ratios in response to metabolic flux for maintaining stable co-culture and balanced growth without manual intervention, was embedded in a two- and three‑strain consortium to produce coniferol and silybin/isosilybin, respectively (Li et al., 2022). Beyond physical monitoring, mathematical modelling, machine learning and artificial intelligence can be united with co-culture systems for large-scale implementation by achieving self-regulated process conditions including medium optimization, microbial proliferation, metabolite biosynthesis, mass transfer and restriction of process disturbances (Lin et al., 2024; Rosero-Chasoy et al., 2021; Thuan et al., 2022; Zhang and Wang, 2016). Lee et al. (2025) developed a computer-based cybernetic system to dynamically control the co-culture of *Pseudomonas putida* and *Escherichia coli* without implementing genetic engineering approaches. The mechanism of this system is based on processing optical density (OD) and fluorescence data obtained from the bioreactor using a mathematical model that captures the growth dynamics of the system in combination with an Extended Kalman Filter. A Proportional–Integral controller compares the estimated composition with the target ratio and automatically adjusts the temperature within the desired range. By leveraging the principle that the two species exhibit different growth rates at different temperatures the system manipulates interspecies competition through temperature modulation, thereby maintaining the population balance at the desired target. This system ensured stabilization of co-culture for 7 days, corresponding to 250 generations. In another study, MicroProphet, a Transformer-based deep learning framework, which utilizes multi-head self-attention and positional encoding to model longitudinal microbiome data without interpolation, was built. The system succeeded in detecting complex interspecies interactions and population dynamics, enabling accurate prediction of microbial abundance trends and the inference of ecological networks (Zhang et al., 2025a, b). By engineering membrane transporters or utilizing more transportable intermediates (e.g., amino acids, organic acids, monosaccharides, some phenolic compounds, alkaloids), enhanced mass transfer in modular co-culture systems can be ensured (Zhang and Wang, 2016; Zhao et al., 2023). Also, examining natural microbial consortia, which exhibit balanced, resilient and productive structure, is able to assist in building desired co-culture systems with higher quality (Jones and Wang, 2018; Liang et al., 2022). Mixed culture systems containing two or more task-specific microorganisms are expected to be further developed and employed for the more efficient implementation of complex biosynthetic processes (Jawed et al., 2019). Lastly, studies utilizing co-cultures containing more than two strains or species to enhance microbial metabolites in foods are relatively fewer than those involving co-cultures of two strains or species (Tables 1–3). Moreover, studies making an increment in metabolites directly in food matrix instead of fermentation medium via co-cultures are significantly scarce, especially in modular co-culture and microbial communication methods (Tables 2 and 3). Therefore, in the future, more systematic and application-oriented studies are required to investigate the potential of co-cultures in food matrices and optimize microbial dynamics for enhanced metabolite production.

In conclusion, this review emphasized how variety of co-culturing strategies affect metabolite synthesis behavior in foods. Microbial interactions, modular co-culture, and microbial communication as co-cultivation strategies can lead to increased production of metabolites in food and consequently the improvement of the food attributes, since these approaches allow microorganisms to influence their metabolic activities reciprocatively. On the one hand, co-cultivated systems bring about advantages such as improved efficiency, reduced costs, greater resilience against perturbations and coping with more complex processes which monoculture could not. On the other hand, the arising drawbacks of system complexity, stability issues and limitations in scaling up for industrial use need to be resolved. The application of the latest advanced technologies such as machine learning, artificial intelligence, high throughput screening and genome editing platforms in future research will facilitate understanding microbial dynamics better, creating more stable systems and challenging these difficulties. To conclude, co-culture approaches have demonstrated great promise for enhancing microbial metabolites in foods; however, more studies adapting advanced technological tools to mixed culture systems to surmount difficulties that come up with co-culturing, and implementing these co-culture strategies, particularly consisting of at least three strains, directly to food matrices for unlocking their full potential will be required in the future.

## Data Availability

The original contributions presented in this study are included in the article. Further inquiries can be directed to the corresponding authors.

## Abbreviations

4-EG: 4-Ethyl-2-methoxyphenol
A1-1: Autoinducer-1
AHLs: *N*-Acyl-homoserine lactones
AI(s): Autoinducer(s)
AI-2: Autoinducer-2
AIP(s): Autoinducing peptide(s)
ATP: Adenosine triphosphate
C: Carbon
CCR: Carbon catabolite repression
CFS: Cell-free supernatant
CL_RK1: *S. cerevisiae* RLAs1654 + *S. cerevisiae* RLAs1642
CL_RK2: *S. cerevisiae* RLAs1654 + *S. cerevisiae* RLAs1643
CL_RK3: *S. cerevisiae* RLAs1654 + *S. cerevisiae* RLAs1642 + *S. cerevisiae* RLAs1643
CoA: Coenzyme A
D-PA: D-pantothenic acid
Fe: Iron ion
GABA: γ-Aminobutyric acid
GAD: Glutamate decarboxylase
HDMF: 4-Hydroxy-2,5-dimethyl-3(2*H*)-furanone
HEMF: 4-Hydroxy-2(or 5)-ethyl-5(or 2)-methyl-3(2*H*)-furanone
HK: Sensor histidine kinase
LAB: Lactic acid bacteria
Lt: *Lachancea thermotolerans* IWBT Y1240
MSG: Monosodium glutamate
N: Nitrogen
NADH: Nicotinamide adenine dinucleotide
NADPH: Nicotinamide adenine dinucleotide phosphate
OD: Optical density
OD_560_: Optical density at 560 nm
*p*-CA: *p*-Coumaric acid
QS: Quorum sensing
RK: Raspberry ketone
Sc: Commercial *Saccharomyces cerevisiae* strain Lalvin EC1118™
WT: Wild type
